# Redo Mitral Valve Replacement After Valve-in-Valve Transcatheter Mitral Valve Replacement

**DOI:** 10.7759/cureus.48438

**Published:** 2023-11-07

**Authors:** Yusuke Tsukioka, Valluvan Jeevanandam

**Affiliations:** 1 Cardiothoracic Surgery, University of Chicago Medicine, Chicago, USA

**Keywords:** trans-catheter aortic valve replacement, transcatheter mitral valve-in-valve replacement, bloodless approach, aortic valve surgery, surgical mitral valve replacement

## Abstract

The rising preference for percutaneous mitral valve-in-valve replacement (ViV TMVR) over redo surgical mitral valve replacement (MVR) is primarily due to its reduced bleeding risk. This report details a bloodless redo MVR performed for mitral stenosis post-ViV TMVR. We present detailed intraoperative findings, including images of the extracted bioprosthetic valves and cardiac anatomy, providing valuable insights into the surgical complexities encountered. The case underscores the importance of meticulous planning and execution in redo MVR, especially in patients with a history of multiple valve interventions. Additionally, this report discusses the potential complications associated with ViV TMVR, contributing to the evolving understanding of this procedure's long-term outcomes. Our findings highlight the need for careful consideration of patient-specific factors and the inherent risks of redo valve surgeries, aiming to improve patient outcomes in complex cardiac cases.

## Introduction

Post FDA approval of percutaneous mitral valve-in-valve replacement (ViV TMVR) in 2017, utilization of ViV TMVR for mitral stenosis (MS) and mitral regurgitation (MR) following mitral valve replacement (MVR) has been on the rise [1]. ViV TMVR benefits include a reliable anchoring point provided by the degenerated bioprosthesis ring and reduced bleeding risk [2-5]. We conducted MVR and aortic valve replacement (AVR) via a median sternotomy for MS after ViV TMVR. As MVR case reports post-ViV TMVR are limited, we present images of the excised valves and the intraoperative cardiac anatomy.

## Case presentation

History of presentation

Our patient, a 67-year-old Jehovah's Witness female with Ehler-Danlos syndrome and rheumatic heart disease, underwent several cardiac procedures for mitral stenosis (MS). In 2015, she had a mitral valve balloon valvuloplasty. At 61 years old in 2017, she chose a bioprosthetic valve (27 mm Mosaic mitral valve, Medtronic, Inc., Minneapolis, MN) over a mechanical one during robotic MVR to avoid warfarin due to bleeding risks and her religious beliefs against blood transfusions. In March 2022, she received a 26-mm Edwards Sapien 3 Ultra valve (Edwards Lifesciences, Irvine, CA) via ViV TMVR for recurrent MS. A month later, severe MS was diagnosed again, leading to her referral to our facility for further surgery.

Investigations

**Figure 1:**
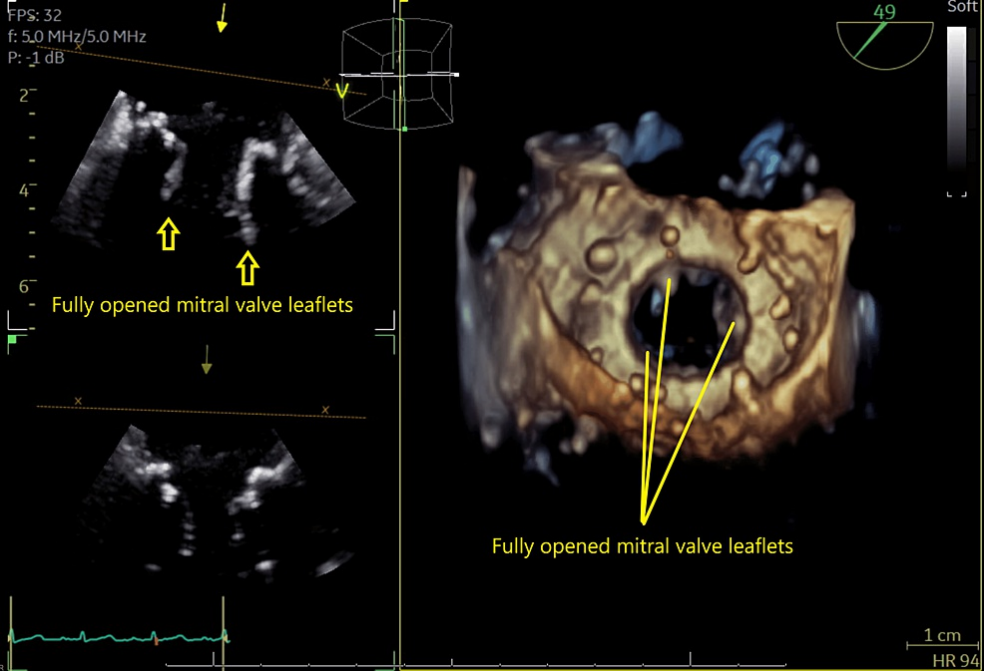
Preoperative echocardiographic depiction of the mitral valve. The Sapien 3 Ultra valve in the mitral position demonstrates preserved leaflet excursion. The yellow arrows and lines show fully opened mitral valve leaflets.

**Figure 2:**
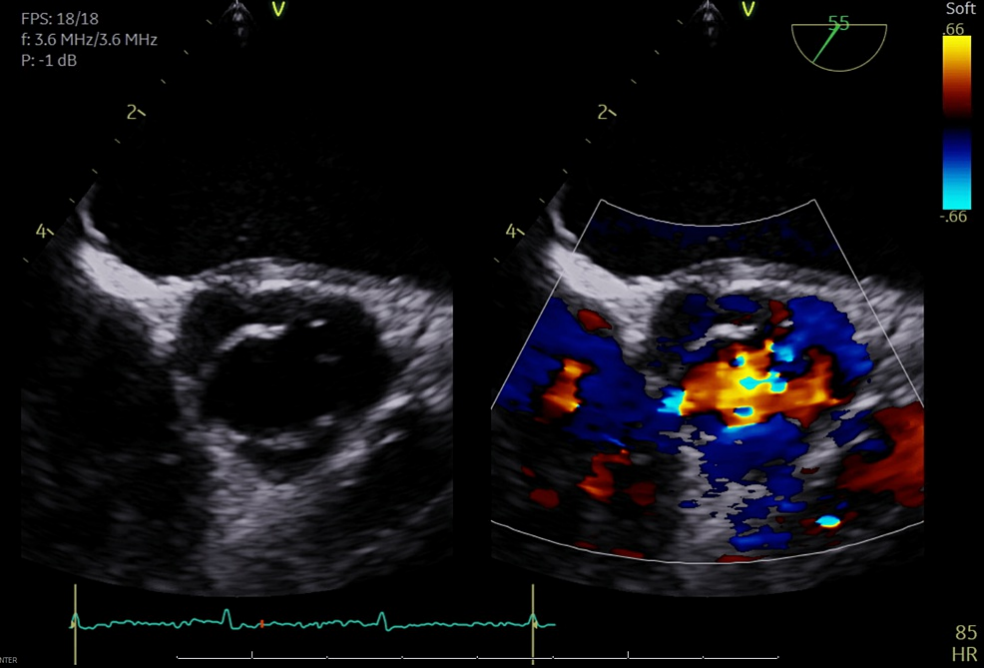
Preoperative echocardiographic depiction of the aortic valve. Preoperative echocardiographic imaging of the aortic valve reveals a trileaflet structure. The echocardiogram shows no significant evidence of aortic stenosis or aortic regurgitation.

During the transesophageal echocardiography (TEE) examination, the left ventricular ejection fraction (LVEF) was observed to be between 50 and 55%. An Edwards Sapien 3 Ultra bioprosthetic valve of 26 mm is situated within a 27 mm Mosaic mitral valve. The observed mean pressure gradient across this valve is between 12 and 15 mmHg. The valve's leaflets are not thickened, and they exhibit preserved excursion, as shown in Figure 1. The internal diameter of the device is 17 mm. Compared to the previously placed surgical prosthesis, this device appears to be fully expanded. It is positioned in the left ventricular outflow tract and extends over the orifice of the aortic valve. The aortic valve consists of three leaflets. Notably, there is no significant aortic stenosis or aortic regurgitation, as depicted in Figure 2.

During the left heart catheterization study, there was evidence of minimal non-obstructive coronary artery disease. In the right heart catheterization study, the following measurements were obtained: Right atrium pressure was 1 mmHg with a mean of 1 mmHg. Right ventricle pressure was recorded as 30/5 mmHg. The pulmonary capillary wedge pressure was 12 mmHg. Pulmonary artery pressure was 30/14 mmHg with a mean of 21 mmHg. The Fick method revealed a cardiac output of 2.03 L/min and a cardiac index of 1.2 L/min/m^2^. Laboratory results showed a hemoglobin (Hgb) level of 13.8 g/dl. The platelet (Plt) count was 131,103 /uL. The creatinine level was 1.14 mg/dL. The estimated glomerular filtration rate (eGFR) was 53 ml/min/1.73 m^2^.

Management

**Figure 3:**
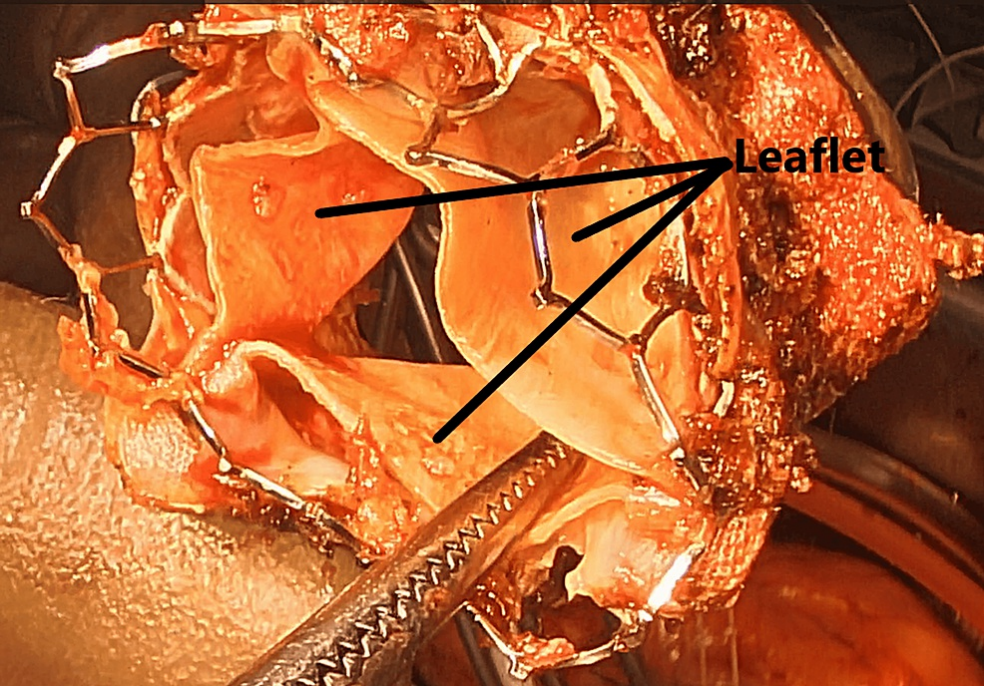
Excised Sapien 3 Ultra mitral valve. Upon examination, there was no evidence of vegetation or pannus formation. Furthermore, the Sapien 3 Ultra valve remained intact, with its leaflets (indicated by the black line) demonstrating appropriate opening and closing dynamics.

**Figure 4:**
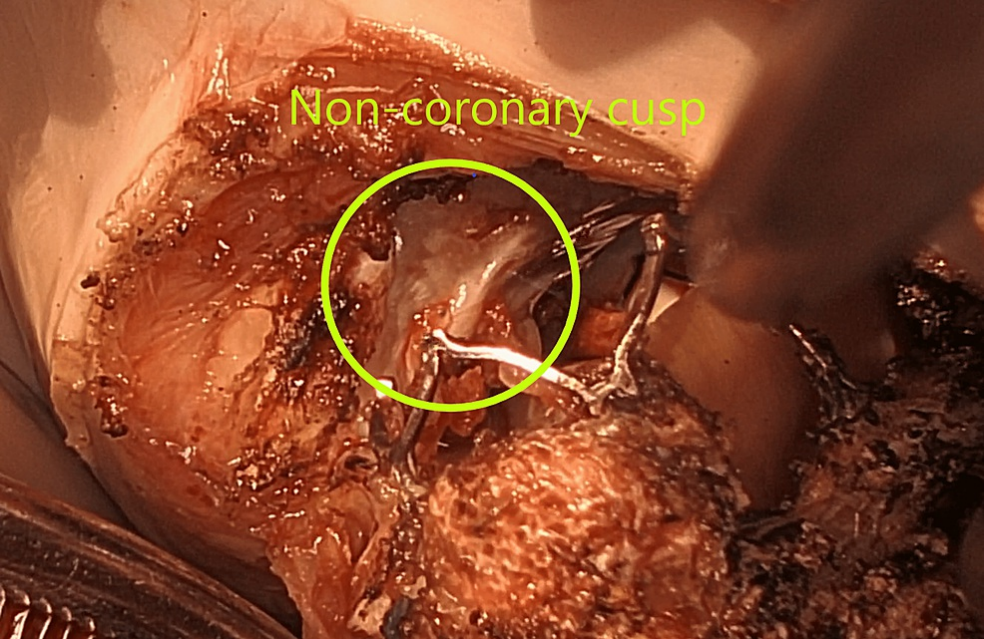
Examination of the non-coronary cusp and mitral annulus post-extraction of the Sapien 3 Ultra valve. The metallic framework of the Sapien valve exhibited robust adhesion to both the non-coronary and left-coronary cusps within the aortic valve and its annulus. Meticulous dissection was employed to separate them; however, partial resection of the aortic valve was necessitated.

**Figure 5:**
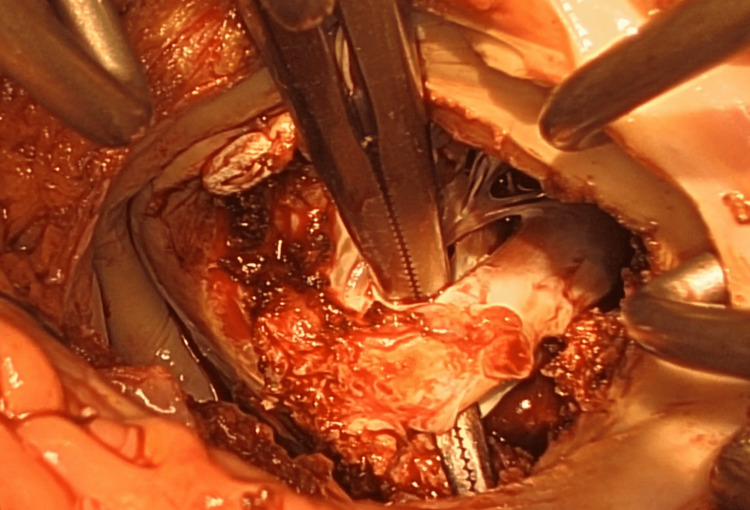
Pathological findings in the papillary muscle. The papillary muscle presented with pronounced calcification and thickening. Concurrently, the chordae exhibited sclerotic changes. These alterations contributed to the outflow obstruction observed in the preceding prosthetic mitral valves, resulting in mitral stenosis (MS) despite the preservation of normal leaflet mobility. A resection was performed on these affected papillary muscles and chordae.

After a median sternotomy and the establishment of cardiopulmonary bypass, the ascending aorta was clamped, and antegrade cardioplegia was administered. After cardiac arrest was obtained, a right atrial incision was made, and a retrograde cannula was inserted into the coronary sinus under direct vision. The mitral valve was reached via a transseptal approach, with no vegetation or pannus and no damage to the Sapien 3 Ultra valve; the Sapien valve leaflets opened and closed well (Figure 3); the metal frame of the Sapien valve was firmly attached to the non-coronary and left-coronary cusps in the aortic valve and annulus. These were carefully separated, but the aortic valve had to be partially shaved off (Figure 4). The papillary muscles were severely calcified and thickened, and the chordae were sclerotic, leading to outflow obstruction of the previous prosthetic mitral valves causing MS despite having normal leaflet mobility. These papillary muscles and chordae were resected (Figure 5). A 33 mm Epic™ Plus Mitral Valve (Abbott Cardiovascular Inc., St. Paul, MN) was secured with 16 pledgeted mattress 2-0 TiCron sutures (Medtronic, Minnesota). A transverse incision was made in the ascending aorta, and observation revealed prolapse of the left- and non-coronary cusps to the left ventricular cavity, which was likely due to the detaching procedure from the Sapien valve. The aortic valve was resected, and a 21 mm INSPIRIS RESILIA Aortic Valve (Edwards Lifesciences LLC, Irvine) was secured with 12 pledgeted mattress 2-0 TiCron sutures.

Postoperative echocardiography showed normal motion of each valve and no regurgitation or stenosis. The mean gradient across the mitral valve after the ViV TMVR procedure was 3 mmHg. The hemoglobin level was 10.3 g/dl postoperatively.

## Discussion

In recent years, an increasing number of ViV TMVRs have been performed for revision of MVRs because of their relatively low risk of bleeding [1]. Ismayl et al. demonstrated that ViV-TMVR yielded superior outcomes compared to redo surgical MVR in patients with degenerated bioprosthetic mitral valves, manifesting in reduced complication rates and abbreviated hospital length of stay, with no notable disparity in mortality rates [3-6]. In our case, ViV TMVR was performed as the patient needed to be operated on without a blood transfusion for religious reasons. However, the patient had severe recurrent MS after ViV TMVR, and surgical MVR through a median sternotomy was chosen to revise the mitral valve.

Although a literature search of PubMed and Embase was conducted, no case reports were found regarding surgical MVR in patients following transcatheter valve-in-valve mitral valve replacement. Therefore, this case report can be considered a valuable contribution to the literature, detailing the anatomical changes around the mitral and aortic valves post-transcatheter ViV MVR.

The 26 mm Sapien 3 Ultra valve had no vegetation or pannus lesions, and the valve excursion was normal. However, the papillary muscles and chordae were significantly thickened and stiffened, suggesting that these thickened papillary muscles in the outflow tract of the small Sapien valve may have increased the pressure gradient as a cause of MS. MS should be considered as one of the complications of ViV TMVR in patients with rheumatic MS. Taha et al. indicated that the incidence of mitral stenosis was higher in ViV TMVR for degenerated prosthetic mitral valves than in ViV TMVR for mitral rings [7].

The metal frame of the Sapien valve was tightly adhered to the aortic valve and annulus. Removing the valve required scraping portions of the aortic valve and annulus. This procedure may have caused aortic regurgitation. We should bear in mind that this type of complication can occur during an open-heart redo MVR after ViV TMVR. 

We assume that aortic regurgitation or stenosis did not occur preoperatively because the adhesion between the metal frames of the Sapien valve and aortic valve/annulus was limited to the area near the valve annulus and did not significantly inhibit the movement of the valve leaflet itself.

Furthermore, when performing a trans-catheter aortic valve replacement (TAVR) after TMVR, it should be remembered that this metal frame may interfere with the guide wire manipulation and deployment of an aortic valve during a TAVR operation.

## Conclusions

This case highlights the potential complications of mitral valve-in-valve replacement (ViV TMVR). Thickened papillary muscles obstructing the outflow of a small Sapien valve can significantly increase the pressure gradient, indicating mitral stenosis (MS) as a possible complication of ViV TMVR. Additionally, the metal frame of the Sapien valve may adhere strongly to the aortic valve and annulus. Its removal can damage the aortic valve, leading to aortic insufficiency. These findings underscore the need for careful consideration of anatomical changes and potential complications when performing ViV TMVR and subsequent interventions.
